# Study claiming target in sepsis with erythromycin has no effect upon mortality and secondary outcomes includes patients with CRRT and RRT

**DOI:** 10.1186/s13054-023-04335-7

**Published:** 2023-02-07

**Authors:** Patrick M. Honore, Sydney Blackman, Emily Perriens, Ibrahim Bousbiat

**Affiliations:** 1 ICU Department, UCL Louvain Medical School, CHU UCL Godinne Namur, Avenue G Thérasse 1, 5530 Yvoir, Belgium; 2 ICU Brugmann University Hospital, ULB University, Brussels, Belgium

Reijnders et al. recently published an article concluding that, in their target trial emulation on critically ill patients with sepsis, they could not demonstrate an effect of treatment with low-dose erythromycin on mortality, secondary clinical outcomes, or host response biomarkers [1]. However, when carefully reviewing the baseline characteristics, we found that 49.8% of the patients in the treated group had acute kidney injury (AKI). Nearly half of critically ill patients—especially those with septic shock—have or developed AKI and 20–25% needed renal replacement therapy (RRT) within the first week of their stay [2]. So in Reijnders’ study, since almost 50% had AKI, we could make the assumption that 20–25% needed RRT or continuous RRT (CRRT). As Reijnders’ study did not provide numbers regarding RRT, this assumption may also overestimate any negative impact on effect estimates. Erythromycin has a molecular weight of 734 daltons making it in theory very easily removable by RRT and CRRT [3]. Although possible in theory, there are almost little to no published data on this issue. CRRT is performed using membranes that have a cut off value of 35–40 kDa; it is therefore logical to assume a considerable portion of erythromycin is eliminated by the CRRT [4]. New highly adsorptive membranes (HAM) are able to adsorb molecules with a molecular weight above 35 kDa, further increasing the removal or erythromycin [5]. Not taking the effect of RRT and CRRT on erythromycin into account can mislead evaluations and conclusions by artificially reducing the level of erythromycin and underestimating its effects in the treatment group [1]. We need to take into account that 75–80% of erythromycin is protein bound, leading to poor clearance by the kidney. Nevertheless, only a study looking into erythromycin clearance could precisely quantify the loss of erythromycin by RRT and the potential impact on the results of the study. If the findings of this new study show that erythromycin is significantly removed by RRT, excluding patients with AKI that may need RRT or CRRT is necessary to avoid potentially underestimating the effects of erythromycin in patients not undergoing RRT or CCRT in the future.

## Data Availability

Not applicable.

## Abbreviations

AKI: Acute kidney injury
RRT: Renal replacement therapy
CRRT: Continuous renal replacement therapy
HAM: Highly adsorptive membranes
